# A High Standard of Attainment the Demand of the Age

**Published:** 1859-03

**Authors:** B. P. Aydelott


### "A HIGH STANDARD OF ATTAINMENT THE DEMAND OF THE AGE."

An Address at the Commencement of the Ohio Dental College, 1859, by the President of the Board of Trustees,

B. P. AYDELOTT, D. D.,

"LOOK ALOFT! LOOK ALOFT!"—was the emphatic direction of a chief officer on the quarter-deck of a tempest-tossed ship, to a man far up in the rigging. He was a green hand, and had been ordered to the mast-head upon duty. Gazing downward from the giddy hight, he was bewildered; a tremor seized him; his grasp was fast relaxing; in a moment more, he would have fallen upon the deck below and been dashed to pieces, or been swallowed up in the yawning gulf. The officer saw his danger, and knew the remedy—the only possible means of escape. Hence the instant, urgent cry, "LOOK ALOFT! LOOK ALOFT!" Nothing could save that imperilled man but turning his eyes away from the fearful depths below and fixing them upon the immeasurable expanse above. A spectacle so sublime would, by a law of his nature, restore him to calmness, and confidence, and strength. The man was saved.

But mark. This scene was witnessed by a youth on the deck. He was poor, uneducated, friendless; and had gone to sea as a means of procuring his bread, and perhaps, also, to gratify a roving disposition and a love of novelty. But the words—look aloft, look aloft—as they fell upon the ear of this forlorn boy, suggested to him a much higher sense than they were intended to convey. "Where am I?" thought he; "what am I doing here? Shall I be content to remain an ignorant, reckless sailor, soon, perhaps, to be shipwrecked and lost at sea? or, still worse, to sink into an early grave the victim of drunkenness and vice, leaving no trace of usefulness, no name of honor behind? forgotten as speedily as the bubble that bursts upon the ocean's bosom?"

From that moment his resolution was formed: "*I will look aloft! I will look aloft!*" was his noble resolve. He applied himself to the pursuit of knowledge; every opportunity was carefully improved; every moment faithfully employed. He advanced—onward and onward—till at last, after years of toil, he reached the Chair of Anatomy in one of our most distinguished Universities.

In a style clear, easy, vigorous, and often very beautiful, he wrote much upon the natural sciences, also, and attained to great eminence in this department.

But he did not long stop here, lofty as his position was. There was something higher which he had not yet reached—something, to which, indeed, he not even looked. Let us follow him, then, a step further in his brief but brilliant history.

For some days he had missed a pupil from his public lectures. He inquired after him from his fellow-students, and found that he was sick. With that sympathy which every professor should feel for each member of his class, he immediately went to see him. He found the young man very ill, nigh unto death. The case was decidedly fatal; and the student, though perfectly aware of his own condition, was yet calm and cheerful.

The Professor was surprised at what he beheld. "Here," thought he, "is a young man, in the bright morn of life—talented—looking forward to high earthly position and much happiness;—but all his prospects are now darkened, his hopes all cut off; and yet he is not at all impatient, or depressed, but calm and cheerful. How can this be? Were I in his situation, I would be, of all men, the most miserable. There is a mystery about this case I can not see through. I must inquire into it." He asked the sick student, how it was he could be so tranquil and cheerful in circumstances so sad? The young man immediately revealed the secret of his happiness—"I am a *Christian*," said he—"a *Christian!*"

The Professor was too much of a philosopher and had too much of common sense, not to be aware that there could be no effect without an adequate cause. He went home, therefore; took his neglected Bible from the shelf, and began to examine its teachings, in order to ascertain, if possible, how the reception of these could produce results so wonderful and benign. Ere long the truth, with heaven's own brightness, shone into his heart, and he himself was made by divine grace to know, in his own soul, what it is to become a new and happy man in Christ Jesus. And thus was he, at last, led up, step by step, to the sublimest realization of that suggestive cry that first aroused him to effort—"Look aloft! look aloft!"

This cry first fell on his ear—a poor sailor boy—and roused him to a career of intellectual improvement—upward and onward, upward and onward—till he found himself in the Professor's Chair. And then, as we have seen, awakened to a conviction, by the facts witnessed at the dying bed of his young friend, that there must be still another hight to which he had not yet attained, he again looked aloft, looked aloft, till "the day-star" dawned upon his heart.

From this time he walked forth, not merely as he had been wont, in all the usefulness and honor of an eminently scientific and literary man, and a richly furnished professional man; but in the still higher attributes of one whose distinguished privilege it was daily to sit together in heavenly places in Christ Jesus;—one, therefore, whose character and life were thus manifestly clothed, in the eyes of all around him, with sweet and most powerful attractions toward that "life and immortality brought to light through the gospel."

But, my young friends, are not your circumstances, in many respects, the same with those of the honored and happy individual here briefly alluded to? You have been taught to "look aloft" to the highest position of professional attainments and usefulness; and, I trust, that you have also been taught that there is an elevation and usefulness which no merely professional or scholarly attainments can achieve. The character of your professors is to me a guaranty that they have endeavored to be faithful to you in this other and most important matter.

But it can not be amiss for one coming to you, as I do, with the weight of years, and of very long professional standing, and clothed also with the highest official responsibility with which the authority of the State could vest him—to ask your serious attention upon this occasion, so momentous in your history, to a subject suggested by the incident we have just briefly sketched. Our subject is then—The necessity of a HIGH STANDARD OF ATTAINMENT.

If we have not altogether mistaken the signs of the times, the world is now calling to you in the tones of a clear, energetic command—"LOOK ALOFT! LOOK ALOFT" First, then, we say—

I. Look aloft *Professionally.*

Time was, we know, when the Dentist was little more than a mere tooth-puller; and too often quite poor at that. But he did well enough then, for no more was expected of him.

Now, however, things are totally changed. He is at this day called to take rank as a Professional man. He must now, therefore, be not only well skilled in his art, but thoroughly furnished in all the sciences which can throw light upon the subjects and the practice of his profession. Anatomy, physiology, pathology, therapeutics, chemistry, materia medica, and more or less of the natural sciences—are all required of him, to understand the evils he is called to grapple with, and the benefits he is expected to confer upon those who put themselves under his care.

No man, whatever readiness he may have acquired in the ordinary routine of the profession, can intelligently practice Dentistry, without more or less acquaintance with these various sciences.

He is not prepared, if unlearned here, to give a reason—the why or the wherefore—for what he does. And should, at any time, as is not seldom the case, the ordinary means and methods fail, he can not, for want of the lights of science, strike out and pursue with confidence a new path to success.

Moreover, the unscientific dentist can never do much to extend the boundaries of his profession, or add to its resources or honors; and hence he can not take a high position among his brethren.

And the Public, too, are quite awake here. They expect to see the practitioner of the dental art well grounded in professional sciences. If, then, in this day you would acquire public confidence and patronage, or retain these, you must look aloft, you must ever keep before you a high standard of professional attainment; you must aim at great things. In a word,--you must win all, or lose all.

But again, the world expect in your profession—

II. A high standard of *general intelligence.*

One of the most striking and cheering characteristics of the day is the almost universal diffusion of knowledge. Education, once shut up to the select few, is now open to all classes. There is scarcely an individual, however humble his position or circumstances, who can not, if he has the capacity and the spirit so to do, rise to the highest rank of scientific and literary attainments. The result is, we have now a hundred really learned men where there were scarcely ten such fifty years ago; and, in point of general intelligence, the present generation is far in advance of the preceding. And there is every reason to believe that this progress will be accelerated with *avalanche* speed.

But if it be so with society at large, what must be the position of the learned professions? These are all expected to take the lead, to occupy an advanced position before the popular mind, if they would continue to benfit society and hold their own honorable standing. The professional man who does not keep ahead of the community, not merely in professional attainments, but in general intelligence also, must quickly be stript of his advantages and sink into contempt. To come up to the average standard of merely professional attainments will not do now. The public demand at this day that the Dentist, as well as the Physician, and the Lawyer, and the Divine, be a man of general information. He must be able to converse well upon the various topics of science and literature, and to appreciate the character and the claims of every department of human knowledge and of the chief laborers in these. If he can not maintain himself in this advanced post of general intelligence, he must suffer all the loss and shame of one who does no honor to his profession and disappoints public expectation.

But there is, once more and thirdly—

III. A still higher sense in which the Dentist must "look aloft." Yes, other and *far higher qualifications* are now required of him than were generally expected even a few years since.

We would, therefore, here, in close, ask your serious attention to these superior excellencies. And in order that you may discern their character and necessity the more clearly and convincingly, let us for a moment just glance backward to the ground over which we have come.

Well, then, my young friends, let us suppose a man to be every thing which we have been describing, every thing which natural talent, literature, science and professional learning and skill can make him; and let us suppose, too, that in the faithful employment of these he has attained to the highest position of professional usefulness and honor. How long will this last? How long? Perhaps twenty, thirty, and, in some rare cases, it may be forty years. And is this all? Will mere intellectual endowments and attainments do no more for him? Must these all soon and forever be buried in the grave with him? Has he brought all his abilities to the work, and then toiled so hard and so long, only to be soon and for ever torn away from his field and see no more of the fruits of his efforts? What waste would this seem to be of mind, of labor, and of learning. And if this be indeed all, what a wreck of human hopes is here! Verily, the more successful our endeavors, the more bitter our disappointment!

But this is not all; and few, if any, now think it so.

> "'Tis immortality deciphers man,
> And opens all the mysteries of his make.
> Without it, half his instincts are a riddle;
> Without it, all his virtues are a dream."

We need not, therefore, say *how*, it is, but it is, nevertheless, the fact that, the world around us have come to such a point of enlightenment as now clearly to see that high moral principle, or, in other words—a *pure heart* is ordinarily essential even to high intellectual effort, and to ensure a successful career, especially in professional life. He who is most under the influence of a *pure heart*, will be sure to possess that calm, hopeful, energetic, far-seeing, lofty-reaching spirit that will best fit him for intellectual exertion and success, and best enable him to labor for and secure the most distinguished professional position. Hence the world will be sure to have most confidence in that man and give him the most substantial evidence of their regard.

But let it always be borne in mind that, this state of heart so neccessary to him who would acquire the highest qualifications, intellectual and professional, for success in life, —this purity of heart, we say, can not be attained if these qualifications and success are all, or even the chief thing the man has in view: he must seek these merely as a means to a higher end. He must pursue science, and literature, and professional knowledge and skill, and success in the use of these, as all subservient to the noblest purpose, the true goal of human aspiration,—the improvement and elevation of his own moral and spiritual nature, the highest good of his fellow men, and the glory of God.

But where can this pure heart—the everwelling spring of true morality, of all human excellence,—where can it be obtained? On what does it rest? We answer—It is God's highest gift; and to Him, therefore, in our deepest need and struggling under our weight of solemn responsibility—to Him must we ever look. It is the fruit, also, of a simple, child-like faith in the great truths of His glorious gospel, and the renewing influences of His Holy Spirit. And, be assured, that he who thus "looks aloft" will more than realize his own sublimest hopes. But with any lower aims, whatever his talents or exertions, his life can not but be, at the most—*a splendid failure.*

Young Gentlemen, you are just about to embark on the ocean of life. All will not be a calm sea before you, and prosperous breezes. You have rocks and storms and breakers to encounter. But there is a desirable port and a glorious land beyond. How unspeakably important that you reach these. Not to do so is to founder and perish. Permit one who has now nearly crossed this perilous sea, and who can not be far from the haven, to give you, before we part, a few sentences of affectionate advice.

First, then, let the word of God always be "the man of your counsel." Read it daily with an humble and teachable heart, ever looking up in prayer for the illuminating and renewing influences of the Holy Spirit. You will in this way never be at a loss to know your duty and do it. "If any man lack in wisdom," says an inspired writer, "let him ask of God, and it shall be given him ; but let him ask in faith."

Again,—Read much. Write much. Teach much ; for to teach others is the way to become the most thorough scholars ourselves. Be continually adding to your library ; count that money well spent which puts a good book upon your shelves.

Further,—be diligent in practice : and recollect that whatever is worth doing at all, is worth doing well. A careless, perfunctory operation can scarcely fail to impair your powers of usefulness as well as your worldly interests ; while every accurate, careful effort will be equally sure to render you the more thorough practioners and give you desired success.

Moreover,—think that day lost whose study and labor have not given you one new idea, or enlarged your capacities for usefulness.

And again,—as we all must be more or less dependent upon each other, try to learn as much as possible from others, and to profit by their influence. For this purpose secure their good will by always exercising good will to them. "He that hath friends," says the wise man, "must shew himself friendly." Therefore, cherish in your heart a real kindness for every human being, and be courteous to all men. It is good to be a general scholar—a man of science and literature ; it is good to be a learned professional man ; and it is best of all to be a true Christian ; but it is good, also, to be a *gentleman.*

And these good things are all before you. They are the excellencies which you must ever reach after, the prize of your high calling. But the work is arduous. Labor, vigilance, wisdom, are all necessary to success. And failure involves tremendous consequences here and hereafter.

But be of good cheer ; quit yourselves like men ; be strong. Blessed be "the Father of Lights,"—we have an infallible directory—"LOOK ALOFT! LOOK ALOFT!" in the highest, truest sense. Yes, look upward in faith to a CRUCIFIED SAVIOR and an OPEN HEAVEN: and such a light will then ever shine upon your path, and such a courage spring up in your heart as can not fail to render you able, useful, honored, happy here; and so fit you at last for the higher excellencies, usefulness, and blessedness of a glorious immortality!

